# Signature for Prostate Cancer Based on Autophagy-Related Genes and a Nomogram for Quantitative Risk Stratification

**DOI:** 10.1155/2022/7598942

**Published:** 2022-07-07

**Authors:** Chenghao Wen, Qintao Ge, Bangshun Dai, Jiawei Li, Feixiang Yang, Jialin Meng, Shenglin Gao, Song Fan, Li Zhang

**Affiliations:** ^1^Department of Urology, The First Affiliated Hospital of Anhui Medical University; Institute of Urology, Anhui Medical University; Anhui Province Key Laboratory of Genitourinary Diseases, Anhui Medical University, Hefei 230022, China; ^2^Department of Urology, The Affiliated Changzhou No. 2 People's Hospital of Nanjing Medical University, Changzhou, Jiangsu, China; ^3^Center for Scientific Research of the First Affiliated Hospital of Anhui Medical University, Hefei 230022, China; ^4^Anhui Provincial Institute of Translational Medicine, Hefei 230032, China

## Abstract

**Background:**

Prostate cancer (PCa) ranks as the most common malignancy and the second leading cause of cancer-related death among males worldwide. The essential role of autophagy in the progression of PCa and treatment resistance has been preliminarily revealed. However, comprehensive molecular elucidations of the correlation between PCa and autophagy are rare.

**Method:**

We obtained transcription information and corresponding clinicopathological profiles of PCa patients from TCGA, MSKCC, and GEO datasets. LAASO analysis was employed to select gene signatures and estimate the autophagy score for each patient. Correlations between the signature and prognosis of PCa were investigated by K-M and multivariate Cox regression analyses. A nomogram was established on the basis of the above results. Further validations relied on ROC, calibration analysis, decision curve analysis, and external cohorts. Variable activated signaling pathways were revealed using GSVA algorithms, and the genetic alteration landscape was elucidated via the oncodrive module from the “maftools” R package. In addition, we also examined the therapeutic role of the signature based on phenotype data from GDSC 2016.

**Result:**

Six autophagy-related genes were eventually selected to establish the signature, including *ULK1*, *CAPN10*, *FKBP5*, *UBE2T*, *NLRC4*, and *BNIP3L*. We used these genes and corresponding coefficients to calculate an autophagy score (AutS) for each patient in this study. A high AutS group and a low AutS group were divided on the mean AutS of the patients. Longer overall survival, higher Gleason score and PSA, and better response to ADT were observed in patients with high AutS. Meanwhile, we found that high AutS PCa was related to more proliferation-associated signaling activation and higher genetic mutation frequencies, manifesting a poor prognosis. A nomogram was constructed based on GS, T stage, PSA, and AutS as covariates. Its discriminative efficacy and clinical value were validated using robust statistical methods. Finally, we tested its prognostic value through two external cohorts and six published signatures.

**Conclusion:**

The autophagy-related gene signature is a highly discriminative model for risk stratification and drug therapy in PCa, and a nomogram incorporating AutS might be a promising tool for precision medicine.

## 1. Introduction

Although prostate cancer (PCa) is generally considered a type of indolent cancer, more than 366,000 estimated deaths are reported annually, and the number ranks as the second leading cause of cancer-related death in men [1]. Benefiting from the widespread use of PSA screening, most PCa cases can be diagnosed earlier and treated effectively with radical resection or radiation. However, 17-31% of these males might still suffer from a high-risk localized or advanced locally advanced disease and would succumb to the disease even after receiving curative treatment [2]. Unfortunately, there are no effective treatments available to solve this predicament to date. Researches on the molecular mechanisms of PCa may provide a promising choice for urologists to screen PCa and risk stratification, optimize personalized treatment and provide novel therapeutic targets.

Autophagy is a highly conserved biological process in all eukaryotes. Currently, three primary distinct types of autophagy have been proposed, including microautophagy, macroautophagy, and chaperone-mediated autophagy (CMA). It mediates cellular degradation and recycling process through delivering cargo to lysosome. Rare studies were conducted for microautophagy for availably limited tools, and limited molecular mechanisms of CMA have been elucidated. Therefore, autophagy often represented macroautophagy solely. In normal mammalian cells, it constitutively occurs at a low level, but once stress happens, it could be further induced to enhance the degradation process and provide more materials and energy for keeping the cells alive [3–6]. However, dysfunction of autophagy would lead to numerous diseases, such as neurodegeneration, myopathies, diabetes, amyotrophic lateral sclerosis, Alzheimer disease, inflammatory bowel disease, and what is more, malignant diseases [7].

Increasing evidence has proven that autophagy plays a complicated role and depends on the context of the tumor [8, 9]. It can suppress tumor progression at the initial stage by protecting the integrity of normal cells and genomic information while promoting tumor progression at a more advanced stage by helping malignant cells escape programmed cell death in hypoxia or nutrient-deficient environments and promoting drug resistance [10–12]. Numerous studies have pointed out the pivotal role of autophagy in PCa. For instance, Chenchu Lin et al. validated autophagy as a promoter in PCa using cell lines, xenografts, and genetic mouse models [9]. Ahamed Saleem et al. found elevated levels of autophagy markers in patients with GS 9 compared with patients with GS 7 and validated the clinical benefit of one type of autophagy inhibitors termed hydroxychloroquine in PCa [13]. *Atg7* is an autophagy-related gene. In an *Atg7* deficiency mouse model, endoplasmic reticulum stress was upregulated, and PCa progression was delayed [14]. These findings encouraged further study on the essential value of autophagy in PCa, from risk stratification to clinical decision-making guidance.

Here, we comprehensively analyzed autophagy-related genes in PCa based on transcription data and clinicopathological profiles from three publicly available databases. With the robust statistical methods, we validated six prognosis-related autophagy genes. Referring to published relevant studies, we built an autophagy-related gene signature and calculated autophagy score (AutS) for each patient based on the expression of six genes and corresponding coefficients [15, 16]. Subsequently, we analyzed the value of AutS in predicting prognosis, risk stratification, and drug susceptibility prediction. Meanwhile, we elucidated the signaling pathways and mutated genes which are responsible for the poor outcome. To facilitate quantitative risk stratification, we also established a nomogram incorporating AutS, Gleason score (GS), PSA, and T stage. Eventually, two external validation cohorts and six published signatures were enrolled to further test the high discriminative ability of AutS.

## 2. Methods

### 2.1. Summary of Enrolled Patients

We collected TCGA-PRAD, MSKCC, GSE70768, and GSE46602 cohorts for the current study. All patients were followed for longer than one month, and they had recorded the features of age, GS, PSA, and pathology T stage and were reserved for the subsequent study. Finally, 431 PCa patients from the TCGA-PRAD cohort, 136 patients from the MSKCC cohort, 108 patients from the GSE70768 cohort, and 27 patients from the GSE46602 cohort were collected. The TCGA-PRAD cohort was used as the training cohort, while the GEO-combined cohort was used as the testing cohort. The GSE54460 and GSE94767 cohorts were also collected as the external validation cohorts. The details of the clinical pathological features are displayed in Table 1.

### 2.2. Preprocessing of the Gene Expression Profiles

The transcriptional profiles corresponding to the abovementioned PCa patient cohorts were downloaded from the Genomic Data Commons (GDC) platform (https://portal.gdc.cancer.gov/) and Gene Expression Omnibus (GEO, https://www.ncbi.nlm.nih.gov/geo/).For the TCGA-PRAD cohort, the number of fragments per kilobase million (FPKM) was computed and converted into transcripts per kilobase million (TPM) and was further log 2 transformed, which showed more similarity to the numbers obtained from microarray analysis. For GEO cohorts, the gene symbol for each cohort was transferred from the probe ID according to the corresponding annotation file from each platform. We also removed the potential cross-dataset batch effect between the three GEO cohorts via the “sva” package along with the empirical Bayes framework [17] and summarized the GEO-combined cohort (Figure S1).

### 2.3. Construction of the Autophagy-Related Gene Signature

We reviewed the literature published in the past five years and collected autophagy-related genes whose function was validated based on experiments. The autophagy genes listed in the Human Autophagy Database (HADb, http://autophagy.lu/), KEGG_REGULATION_OF_AUTOPHAGY, and WP_AUTOPHAGY gene sets were also collected. Autophagy genes associated with PCA recurrence-free survival (RFs) were identified by univariate Cox regression analysis of the combined TCGA-PRAD and GEO cohorts. Least absolute shrinkage and selection operator (LASSO) Cox regression, which reveals the linear relationship between the features and targets, was conducted to select stable prognostic autophagy genes and support the construction of the prognostic signature with the corresponding index.

(1) $$\begin{array}{c} \text{Autophagy score}=\overset{n}{\underset{i=1}{\sum }}\left[\text{coef}\left(\text{selected gene}\right)*\text{Expression}\left(\text{selected gene}\right)\right]. \end{array}$$

Index of selected genes was generated through the TCGA-PRAD cohort which is used as a training cohort. The autophagy score (AutS) of patients in the GEO-combined cohort was calculated according to the above formula, as well as the external validation GSE54460 and GSE94767 cohorts.

### 2.4. Construction and Validation of the Nomogram

The prognostic nomogram was built by independent prognostic factors identified by multivariate Cox regression analysis. It can predict the recurrence probability individualized, and this quantitative tool provided for urologists was established by the R package “regplot.” The model goodness of fit was estimated by calibration curves. Decision curve analysis and clinical impact curves quantified the net benefits at different threshold probabilities to determine the clinical usefulness of the nomogram by using the R packages “rms” and “rmda.” The receiver operating characteristic (ROC) curve and the area under the curve (AUC) were used to assess the predictive function of the nomogram [18].

### 2.5. Evaluation of Molecular Function

The variations in the pathway activities among high-AutS and low-AutS groups were assessed by gene set variation analysis (GSVA) with the “GSVA” R package. The 50 hallmark gene sets were obtained from MSigDB [19, 20]. The genetic mutations of PCa patients were also enrolled from the GDC by the “TCGAbiolinks” package and further visualized via the “maftools” R package [21]. The drive gene of PCa was also evaluated by the oncodrive module from the “maftools” R package.

### 2.6. Evaluation of Response to Chemotherapy and Androgen Deprivation Therapy (ADT)

For chemotherapy and ADT, the data of drug sensitivity and phenotype were used to predict the therapeutic response via the R package “MOVICS” from GDSC 2016 (https://www.cancerrxgene.org/) [22]. The estimated inhibitory concentration (IC50) was compared among the patients with high-AutS and low-AutS. We also downloaded the RNA sequence data from GSE150475, GSE69249, and GSE88210, which contain the samples that received DMSO or androgen receptor inhibitors, to confirm the predictive value of autophagy for therapy sensitivity. Moreover, we also searched for potential therapeutic new drugs through the Gene Set Cancer Analysis (GSCA) online website (http://bioinfo.life.hust.edu.cn/GSCA).

### 2.7. Collection of Proposed PCa Prognosis Signatures

We compared the AUC values depending on the autophagy signature and several proposed signatures to determine the prognostic ability of the autophagy-related gene signature. Zhang et al. [16] reported a PCSS signature based on 13 genes linked with tumor stem-like features. Liu et al. [23] also constructed a stem cell-associated gene set signature to predict the RFS of PCa, which contains 13 genes as well but is totally different from Zhang's. The CCP score was calculated as the ratio of the mean expression value of 31 cell cycle proliferation genes to the mean expression value of 15 housekeeping genes [24]. Yang et al. [25] identified a 28-gene hypoxia-related prognostic signature. Cheville et al. [26] only used TOP2A and CDH10 to predict the RFS of the prognostic signature. Glinsky et al. [27] identified a signature with 11-gene in transgenic mouse models of PCa and cancer patients; this signature always showed a stem-cell-like expression profile. Based on our preprocessed gene expression profile, the risk score for each registration signature was calculated according to the index mentioned in the corresponding article.

### 2.8. Statistics

We use R (Version: 4.0.2) to execute all statistical analyses. K-M curves were generated by the log rank test to analyze survival rates for patients with different detection methods. ROC analyses performed by the R package “pROC” were wielded to examine the prediction efficiency of the autophagy-related gene signature. The time-dependent prognostic value of the autophagy signature and proposed signatures was illustrated and compared by the “timeROC” R package. A two-tailed *P* value <0.05 was recognized as statistically significant. Hazard ratios (HRs) and 95% confidence intervals (95% CIs) for RFS were estimated via Cox proportional hazards regression. A forest plot was exhibited to assess the prognostic value of the autophagy-related gene signature and other features by the function “ggforest” in the R package “surviminer.”

## 3. Result

### 3.1. Construction of an Autophagy-Related Gene Signature and Prognostic Analysis

**Figure 1:**
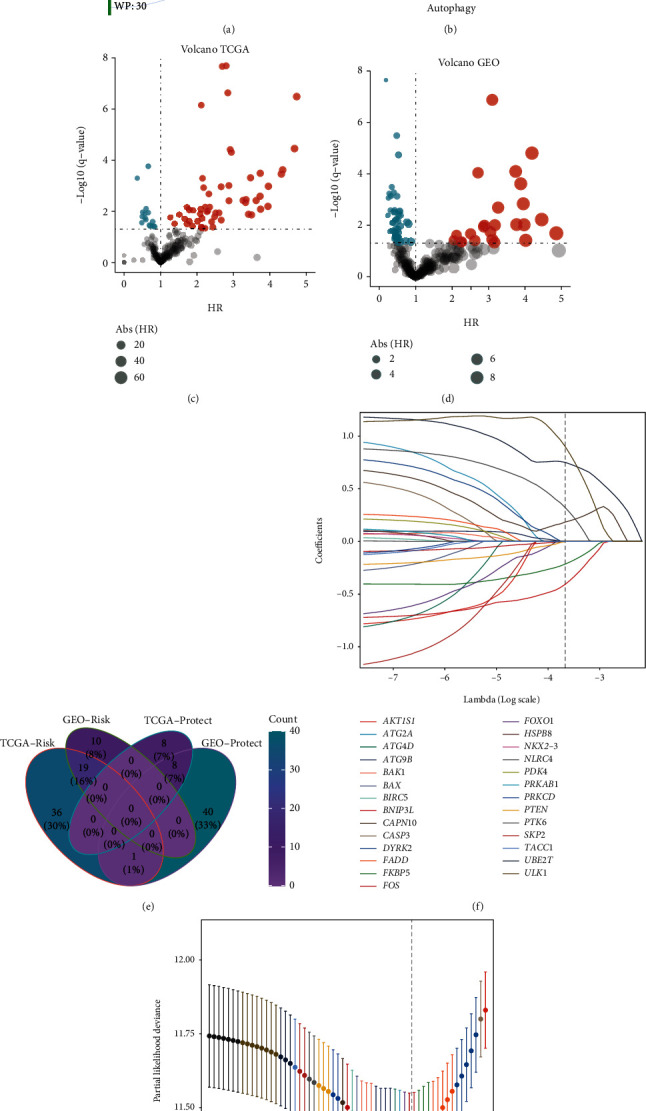
Selection of prognosis-related autophagy genes. (a) Total autophagy-related genes collected in this study. (b) Risky and protective autophagy-related genes in GEO cohort; risky genes are highlighted with red, and protective genes are highlighted with blue. (c) Risky and protective autophagy-related genes in TCGA-PRAD cohort; risk genes are highlighted with red, and protective genes are highlighted with blue. (d) Autophagy-related genes shared between GEO-combined and TCGA-PRAD cohorts. (e) Intersection of risky genes and intersection of protective genes among two cohorts. (f) Plots for LASSO coefficients under different penalty values. (g) Plot for the penalty term in cross-validation.

**Figure 2:**
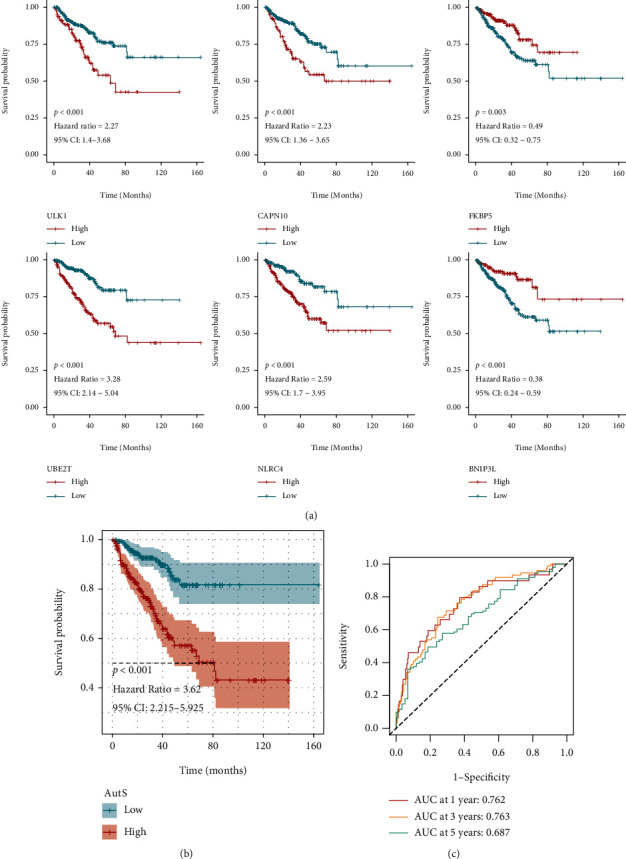
Survival analysis of autophagy-related gene signature. (a) K-M curves for *ULK1*, *CAPN10*, *FKBP5*, *UBE2T*, *NLRC4*, and *BNIP3L*. (b) K-M curves for autophagy-related gene signature. (c) ROC analysis for autophagy-related gene signature at 1 year, 3 years, and 5 years.

We obtained 410 autophagy-related genes, 124 from prior literature, 222 from the HADb database, 34 from KEGG_REGULATION_OF_AUTOPHAGY, and 30 from the WP_AUTOPHAGY gene sets. After merging duplicated genes, 352 genes were enrolled (Figure 1(a)). To make the results comparable, we intersected 352 genes with GEO-combined and TCGA-PRAD cohorts to obtain genes shared by them (*n* = 300, Figure 1(d)). We investigated the correlations between 300 genes and the prognosis of PCa in GEO-combined and TCGA-PRAD cohorts. Risky genes and protective genes were flagged and showed in volcano maps (Figures 1(b) and 1(c)). 19 risky genes and 8 protective genes shared by both two cohorts showed in a Venn diagram (Figure 1(e)). Then, in order to screen out the most suitably prognostic genes, LASSO regression analysis was performed based on the 27 genes, and six autophagy-related genes were eventually selected to establish an autophagy-related gene signature, including *ULK1*, *CAPN10*, *FKBP5*, *UBE2T*, *NLRC4*, and *BNIP3L* (Figures 1(f) and 1(g)). *BNIP3L* and *FKBP5* represented protective factors, yet ULK1, *CAPN10*, *UBE2T*, and *NLRC4* represent risk factors in PCa (Figure 2(a)). With the corresponding coefficients of the six genes, AutS was calculated the following formula: AutS = ULK1 expression∗0.901 + UBE2T expression∗0.751 + CAPN10 expression∗0.184 + NLRC4 expression∗0.322 + FKBP5 expression∗(−0.214) + BNIP3L expression∗(−0.407). A negative correlation between the AutS and OS of PCa patients was observed (*R* = −0.12, *P* = 0.012, Figure S2A). Subsequently, we divided all patients in TCGA-PRAD cohort into high-AutS (*n* = 216) and low-AutS (*n* = 215) subgroups with the mean value of AutS. Different distributions of clinicopathologic features were found in the two newly defined subgroups, including survival status (*P* < 0.001), T stage (*P* < 0.001), GS (*P* < 0.001), PSA (*P* = 0.019), and age (*P* = 0.033) (Figure S2B). K-M curve revealed that the OS of patients with high AutS was significantly longer than those with low AutS, with a *P* value less than 0.001 (95% CI: 2.215-5.925) and an HR of 3.62 (Figure 2(b)). To verify the discriminative ability of AutS, ROC was performed. The AUC values were 0.762, 0.763, and 0.687, respectively, at 1, 3, and 5 years, implying good discriminative efficiency of the AutS (Figure 2(c)). As shown in Figure S3, we divided the whole samples in TCGA-PRAD cohort into eight clusters with distinct clinicopathological features and conducted a survival analysis in each cluster. It was found that AutS also showed satisfactory prognostic prediction potential in age ≤65 (*P* < 0.001), age>65 (*P* = 0.036), T3+4 stage (*P* < 0.001), Gleason 6 + 7 (*P* = 0.014), and PSA ≤10 (*P* <0.001) clusters. Unfortunately, no statistical significance was found in T2 stage (*P* = 1.03), Gleason 8 + 9 + 10 (*P* = 0.056), and PSA>10 (*P* = 0.999) clusters, which might be explained by the small sample size in these clusters.

### 3.2. Construction of a Nomogram Risk Model Incorporating the Autophagy Score

**Figure 3:**
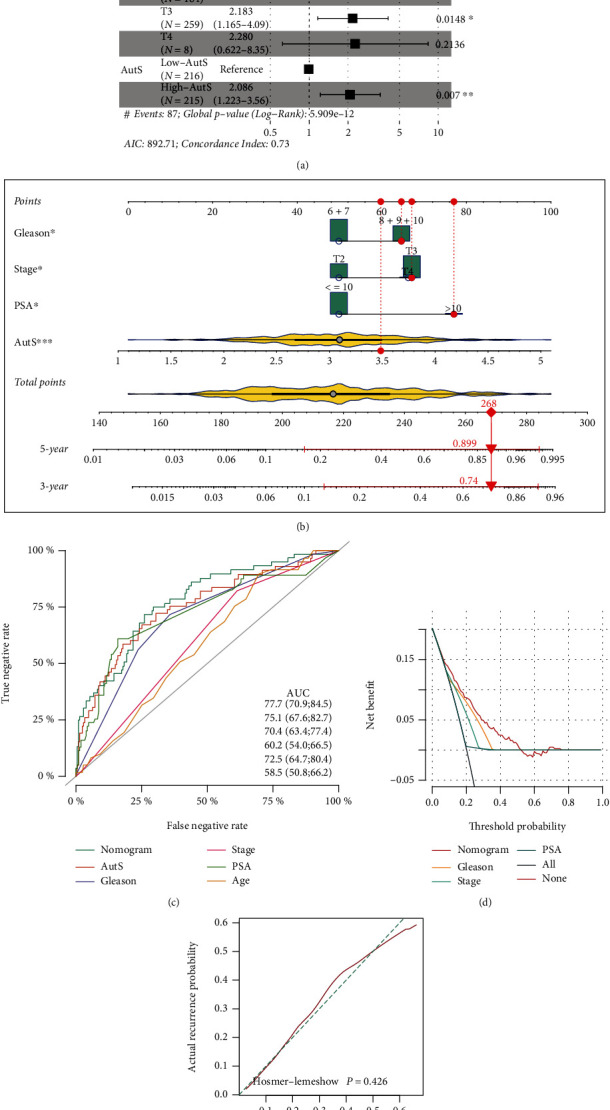
Multivariate Cox regression analysis and nomogram risk model establishment. (a) Independent prognosis analysis via multivariate Cox regression. If the whole segment is located on the right, the corresponding predictor is deemed as a risky factor for PCa or a protective factor which is independence. (b) Establishment of a novel nomogram risk model based on GS, T stage, PSA, and AutS. For a given patient with a GS>7, T3 or T4 stage, PSA>10, and AutS =3.5, we obtained points for each predictor on the points line, calculated the sum of overall points, and found its location on the total points line at the bottom, which was 268. Then, a straight line was delineated from the total point to the 3-year and 5-year lines, and the corresponding points of intersection represented 3-year and 5-year recurrence rates, which were 0.74 and 0.899, respectively. (**c**) Comparison of discriminative performance between the nomogram and AutS, GS, PSA, T stage, and age in TCGA-PRAD cohort. (d) Clinical value assessment for nomogram via DCA. (e) Calibration analysis for nomogram. Dashed line represented observed value and solid line represented predictive value, and a *P* > 0.05 implied a good agreement between observation and prediction values.

Multivariate Cox regression analysis indicated that PSA (*P* = 0.04, HR = 2.565, 95% CI: 1.013-6.50), GS (*P* < 0.001, HR = 2.529, 95% CI: 1.486-4.3), T3 stage (*P* = 0.0148, HR = 2.183, 95% CI: 1.165-8.35), and AutS (*P* = 0.007, HR = 2.086, 95% CI: 1.223-3.56) were independently prognostic risk factors for PCa (Figure 3(a)). Based on the results, we established a nomogram risk model combining GS, T stage, PSA, and AutS for the quantitative prediction of 3-year and 5-year recurrence rates (Figure 3(b)). ROC analysis demonstrated that AUC values of nomogram, AutS, GS, T stage, PSA, and age were 0.777 (95% CI: 70.9-84.5), 0.751 (95% CI: 67.6-82.7), 0.704 (95% CI: 63.4-77.4), 0.602 (95% CI: 54.0-66.5), 0.725 (95% CI: 64.7-80.4), and 0.585 (95% CI: 50.8-6.2) (Figure 3(c)). These results implied that the nomogram had the highest predictive efficiency among them. DCA of the nomogram was employed to evaluate the clinical benefit of it. At a threshold probability from 0 to 0.5, this nomogram presented a higher clinical net benefit than other policies, such as GS, T stage, and PSA (Figure 3(d)). The *P* value in the calibration analysis was 0.426, indicating the goodness of fit between prediction and observation values (Figure 3(e)). Taken together, results mentioned above suggested that the nomogram was almost equal to an ideal risk model.

### 3.3. Validation in the GEO-Combined Cohort

**Figure 4:**
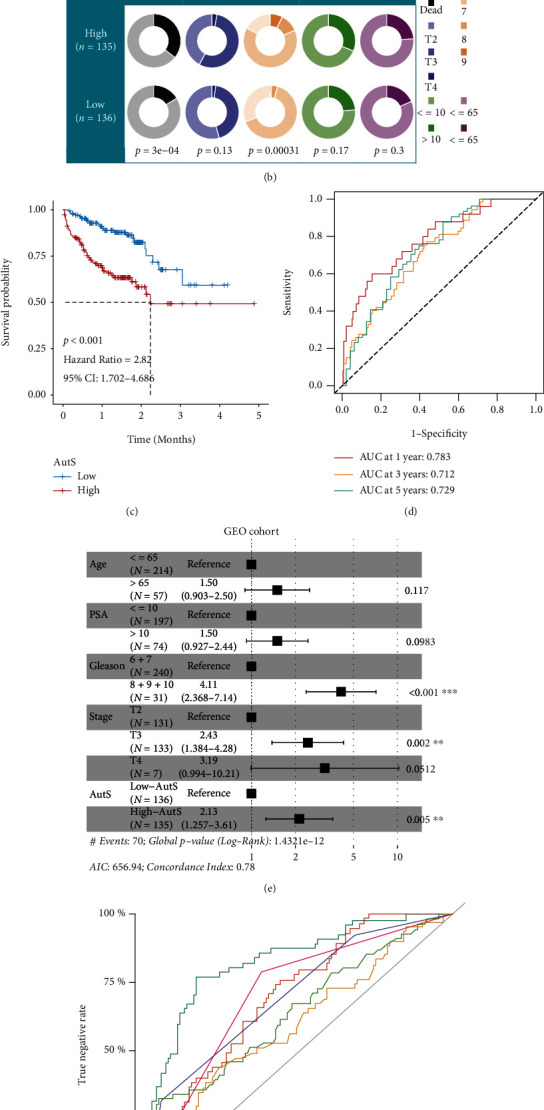
Validation in GEO-combined cohort. (a) Negative association between AutS and OS of PCa in GEO-combined cohort. (b) Clinicopathological distribution among high-AutS and low-AutS groups. (c) K-M analysis for autophagy-related gene signature. (d) ROC analysis for autophagy-related gene signature at 1 year, 3 years, and 5 years. (e) Validation of independent risk factors in PCa via multivariate Cox regression analysis based on the data from GEO-combined cohort. (f) Comparison of discriminative performance between the nomogram and AutS, GS, PSA, T stage, and age.

In the GEO-combined cohort, the prognostic value of *ULK1*, *CAPN10*, *FKBP5*, *UBE2T*, *NLRC4*, and *BNIP3L* was also validated separately (Figure S4). The same formula was employed to calculate the AutS for each patient in the GEO-combined cohort. It was negatively correlated between the AutS and OS of PCa (*R* = −0.22, *P* = 0.00035, Figure 4(a)). According to the average AutS of patients in the GEO-combined cohort, we divided them into high-AutS (*n* = 135) and low-AutS subgroups (*n* = 136). Significantly higher mortality rates (*P* = *P* < 0.001) and GS (*P* = 0.00031) were observed in the high-AutS subgroup than in the low-AutS subgroup, but the difference in T stage (*P* = 0.13), PSA (*P* = 0.17), or age (*P* = 0.3) was not significant (Figure 4(b)). Survival analysis confirmed that high-AutS patients had shorter OS than low-AutS patients (*P* = 0.001, HR = 2.82, 95% CI: 1.702-4.686), which was also examined at 1 year, 3 years, and 5 years via further ROC analysis with AUC values of 0.783, 0.712, and 0.729, respectively (Figures 4(c) and 4(d)). Multivariate Cox regression analysis conducted in the GEO-combined cohort confirmed that GS (*P* < 0.001, HR = 4.11, 95% CI: 2.368-7.14), T3 stage (*P* = 0.002, HR = 2.43, 95% CI: 1.384-4.28), and AutS (*P* = 0.005, HR = 2.13, 95% CI: 1.257-3.61) were independent risk factors for PCa, but no PSA (*P* = 0.0983, HR = 1.50, 95% CI: 0.927-2.44), which was slightly different from the results in the TCGA-PRAD cohort. The predictive performance of our nomogram was further estimated by ROC analysis, and the AUC values of AutS, GS, T stage, PSA, and age were 0.836 (95% CI: 77.0-90.2), 0.709 (95% CI: 63.3-78.5), 0.708 (95% CI: 64.0-77.7), 0.717 (95% CI: 64.8-78.6), 0.647 (95% CI: 55.8-73.7), and 0.598 (95% CI: 50.9-68.7), respectively (Figure 4(f)). Obviously, the combination of AutS, GS, T stage, and PSA resulted in a significantly increased AUC as compared to them alone. Similarly, we assessed the predictive accuracy of AutS in patients with the same clinicopathological features. It also performed well in most context which included age ≤65 (*P* = 0.006), age>65 (*P* = 0.005), T3+4 stage (*P* < 0.001), Gleason 6 + 7 (*P* = 0.018), Gleason 8 + 9 + 10 (*P* = 0.029), PSA ≤10 (*P* = 0.001), and PSA>10 (*P* = 0.018) clusters, but except T2 stage cluster (*P* = 0.629) (Figure S5).

### 3.4. High-AutS PCa Had Numerous Activated Proliferation Pathway and Higher Sensitivity to Drug Therapy

**Figure 5:**
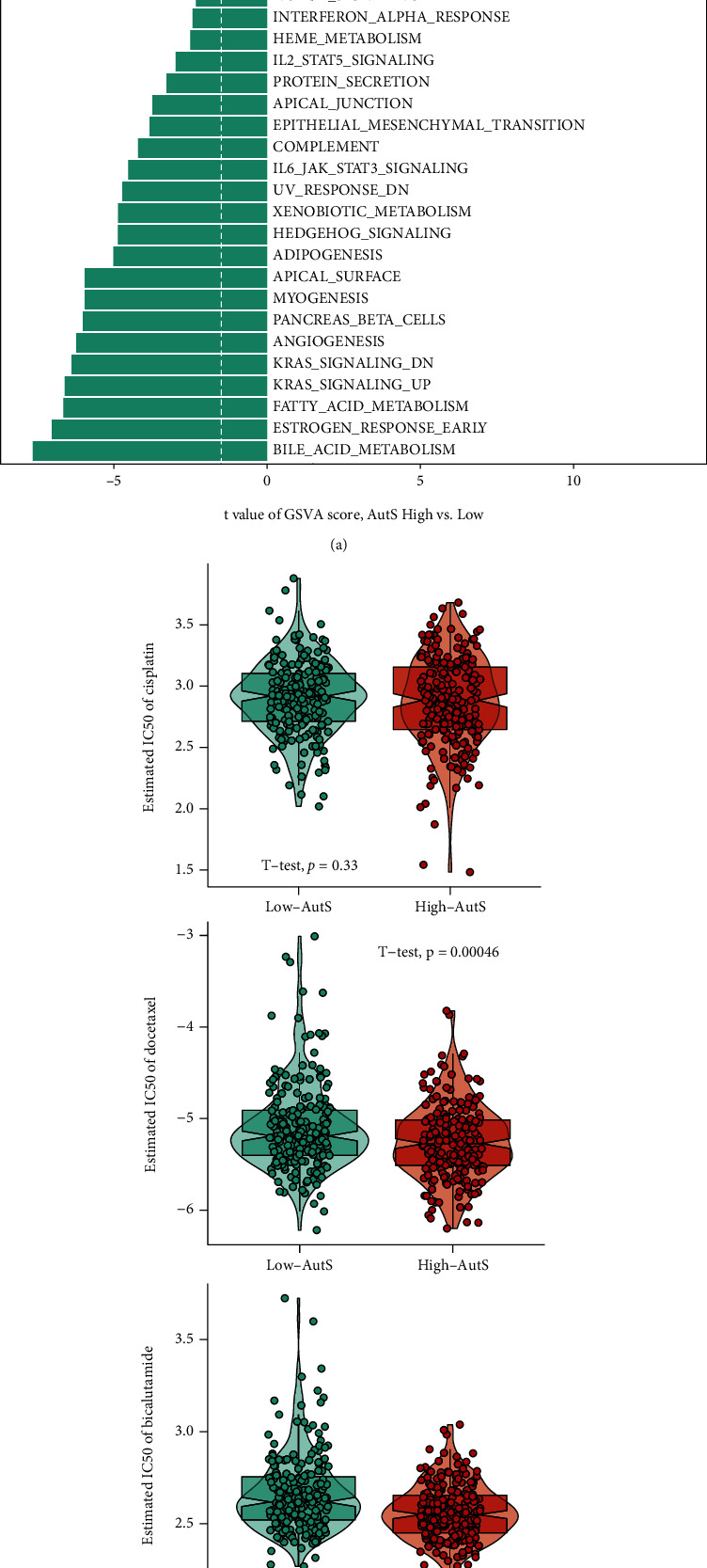
Metabolic pathway enrichment analysis and prediction of response to chemotherapy. (a) Different activated pathways between high-AutS and low-AutS groups. (b) Comparison of susceptibility to cisplatin, docetaxel and bicalutamide among two groups. IC50 was set as the efficacy evaluation indicator. High IC50 represented a bad efficacy. (c) Decreased AutS in LNCaP cells after treatment with bicalutamide and decreased AutS in PCa cells after treatment with enzalutamide. Gene expression data of bicalutamide-treated LNCaP cells was from GSE150475. Gene expression data of enzalutamide-treated PCa cells was from GSE69249 datasets. The label “DMSO” on the *X* axis represented the control groups, and the labels “Bicalutamide” and “enzalutamide” represented the treatment groups.

GSVA was performed to investigate patterns of signaling pathway activation among AutS subgroups. As shown in Figure 5(a), a series of proliferation-associated pathways are activated in the high-AutS group. The G2 M checkpoint, E2F targets, mitotic spindle, and MYC targets V1 and V2 pathways are involved in the cell cycle and have been marked as proliferation-associated pathways in the MSigDB [28]. In addition, mTORC1 signaling was proven to promote tumor progression by regulating metabolic reprogramming, such as aerobic glycolysis, lipogenesis, and purine and pyridine synthesis [29]. The peroxisome was proven to promote tumor progression by regulating fatty acid oxidation, and the oxidative phosphorylation pathway generated ATP to provide energy for tumor cells [30, 31]. These findings suggested that activation of the G2 M checkpoint, E2F targets, mitotic spindle, MYC targets V1 and V2 pathways, mTORC1, peroxisome, and oxidative phosphorylation pathways might be responsible for the poor clinical outcome of PCa, and these pathways may serve as novel therapeutic targets.

Based on the data from GEO and GDSC 2016 databases, we assessed the predictive power of AutS for response to chemotherapy and ADT. High-AutS group presented a lower IC50 in both docetaxel (*P* = 0.00046) and bicalutamide class (*P* < 0.001), but not cisplatin (*P* = 0.33), which suggested a high sensitivity to docetaxel and bicalutamide (Figure 5(b)). On the other hand, a decreased AutS was observed in PCa patients treated with bicalutamide (*P* = 0.032) or enzalutamide (*P* = 0.0019) compared to those received DMSO (Figure 5(c)). It reconfirmed that high-AutS PCa was more sensitive to chemotherapy and ADT. Therefore, the autophagy-related gene signature might be a potential biomarker for chemotherapy and ADT. We also searched the new drugs from the GSCA dataset, a total of 29 components were identified that functioned in the clinical treatment of patients with high AutS (Table 2).

### 3.5. Investigation of Molecular Mechanisms

**Figure 6:**
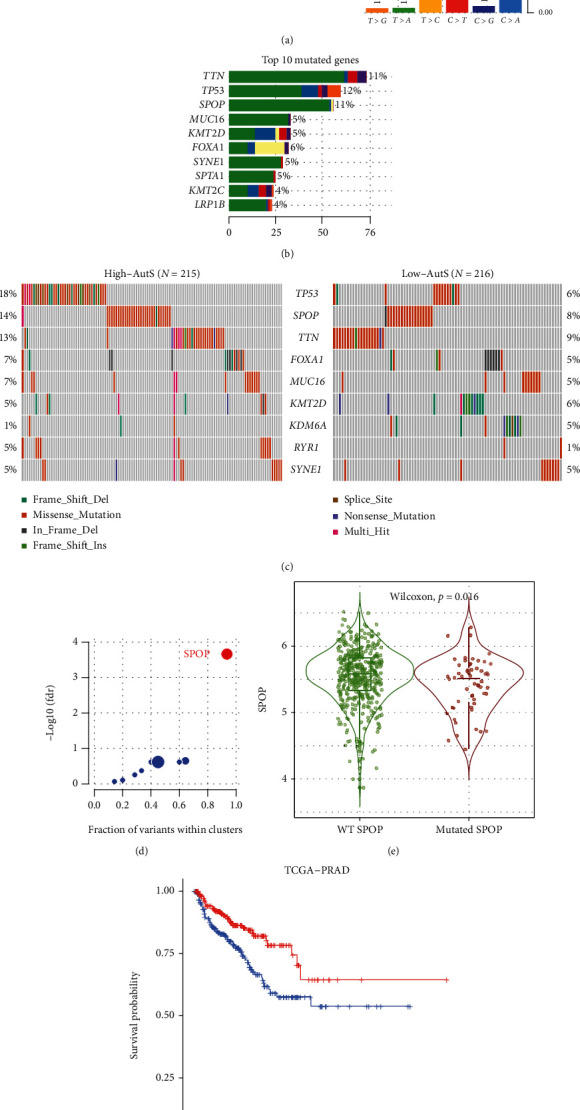
Genetic alteration landscape between high-AutS and low-AutS groups. (a) The types of gene mutation and the abundance of different base pairs in PCa. (b) The top ten mutated genes in PCa. (c) Comparison of the genetic alteration landscape between high-AutS and low-AutS groups. (d) Analysis of the driver gene in PCaE. Comparison of expression level of SPOP between WT *SPOP* type and mutated *SPOP* type. (f) K-M curves for SPOP.

We investigated the molecular alteration landscape among the AutS groups. The most frequent variant type in PCa was missense mutation. At the group level, the most frequent variant type was single-nucleotide polymorphisms (SNPs) (Figure 6(a)). The top ten mutated genes are listed in Figure 6(b), including *TP53* (12%), *TTN* (11%), *SPOP* (11%), *FOXA1* (6%), *MUC16* (5%), *KMT2D* (5%), *SYNE1* (5%), *SPTA1* (5%), *KMT2C* (4%), and *LRP1B* (4%). Compared with low-AutS group, there were more frequent genetic alterations in the high-AutS group. For instance, *TP53* was the most frequent genetic mutation in the high-AutS group, accounting for 18%, while it was only 6% in the low-AutS group; *TTN* was the most frequent genetic mutation in the low-AutS group, accounting for 9%, while it was 13% in the high-AutS group (Figure 6(c)). As identified by the “maftools” R package, *SPOP* was considered as the cancer driver gene (Figure 6(d)). Further study manifested that *SPOP* mutation would inhibit itself expression (*P* = 0.016, Figure 6(e)), and low expression of *SPOP* would result in a shorter OS (Figure 6(f)), which was consistent with prior results that high-AutS was tightly related to a higher *SPOP* mutation frequency and poorer prognosis. Taken together, evidences above manifested *SPOP* mutation events contributed to a poor clinical outcome of PCa.

### 3.6. Verification in an External Cohort and Performance Comparison with Proposed Signatures

**Figure 7:**
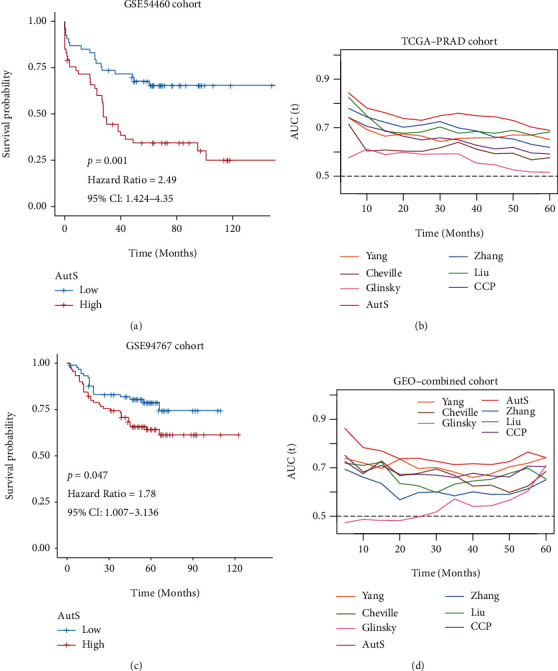
External cohort validation and comparison of discriminative efficiency between autophagy-related gene signature and six published signatures. (a) K-M curves for autophagy-related gene signature in GSE54460 cohort. (b) Comparison of discriminative efficiency between autophagy-related gene signature and six proposed signatures via time-dependent ROC analysis in TCGA-PRAD cohort. (c) K-M curves for autophagy-related gene signature in GSE94767 cohort. (d) Comparison of discriminative efficiency between autophagy-related gene signature and proposed signatures via time-dependent ROC analysis in GEO-combined cohort.

To further verify the predictive efficiency of the autophagy-related gene signature, we reproduced the classification process in GSE54460 (Figure 7(a)) and (Figure 7(c)) GSE94767 cohorts. As the results showed, AutS also performed well in distinguishing good and bad clinical outcomes in both the GSE54460 (*P* = 0.001, HR = 2.49, 95% CI: 1.424-4.35) and GSE94767 (*P* = 0.047, HR = 1.78, 95% CI: 1.007-3.136) cohorts. In addition, there are large amounts of prognostic signatures of PCa have been proposed, but their discriminative abilities are different. To further evaluate our model, we employed time-dependent ROC analysis to compare the discriminative ability of AutS with six published signatures. It was confirmed that AutS had stronger predictive ability than the six published prognostic signatures mentioned above, especially in predicting the recurrence rate within 5 years (Figures 7(b) and 7(d)).

## 4. Discussion

PCa originated from multifocal tumor foci and is famous for high heterogeneity of clinical, molecular, and prognostic characteristics. The diversity of unique molecular alterations between different patients poses challenges for PCa diagnosis and managements. Risk stratification and prognostic prediction schemes based on GS, PSA, and T stage cannot meet all the needs in clinical [32]. Herein, recent researches shifted towards molecular subtyping for PCa for a better understanding of such malignancy. Comprehensive molecular profiling has elucidated a mass of core molecular alterations in PCa and their unique impact on prognosis, especially autophagy-related genes [33].

A total of 352 autophagy-related genes from publicly available datasets and published literature were appraised in this study. After row data processing and LASSO Cox regression analysis, we filtered out six autophagy-related genes and established a prognostic model. We divided whole patients in the enrolled cohort into high-AutS group and low-AutS groups based on the AutS of each patient. K-M analysis revealed that high-AutS patients had a shorter OS, and this result was validated in both ROC analysis and external cohorts, indicating that this prognostic model is robust. Moreover, we found that the AutS served as an independent risk factor in PCa, which was also validated in the GEO-combined cohort. We also investigated the association between AutS and clinicopathological features, and the results presented the high-AutS group had obviously higher GS, T stage, and PSA levels than the low-AutS group, suggesting that AutS is able to distinguish “high-risk” and “low-risk” PCa traditionally defined derived from GS, T stage, and PSA. As a result, mortality was higher in the high-AutS group than in the low-AutS group. On the other hand, elevated autophagy markers were observed in GS 9 PCa compared with GS 7 PCa [13]. Therefore, there is no doubt that a high AutS represents a poor clinical outcome.

Androgen receptor (AR) is always considered a driver leading to the oncogenesis, growth, and metastasis of PCa [34]. They drive the development of PCa throughout the whole natural disease history, even at the relapsed stage, which is termed metastatic castration-resistant PCa (mCPRC). Currently, ADT is the standard first-line therapy for metastatic PCa. It can effectively slow the development of PCa initially but often fails within 2-3 years, and consequently, PCa progresses to mCPRC [9]. Understanding how AR drives PCa progression will help us to find breakthroughs to overcome the inevitable resistance to ADT. Regrettably, the molecular mechanism underlying this resistance remains elusive. Recent studies confirmed AR-mediated autophagy as a potential way to promote PCa cell growth. Alicia M. Blessing et al. detected that androgen upregulated the expression of four essential autophagy genes, including *ATG4B*, *ATG4D*, *ULK1*, and *ULK2*. They validated the necessary role of ATG4B, ATG4D, ULK1, and ULK2 for the proliferation and metastasis of PCa [35]. *CAMKK2* is a downstream target gene of AR in PCa. AMPK and ULK1 are regulators of autophagy, and Chenchu Lin et al. reported that CAMKK2 promotes PCa progression and mCRPC growth via AMPK-ULK1 signaling [9]. These findings indicated that autophagy markers might be potential predictors for ADT. In our study, we observed that high-AutS PCa was more sensitive to bicalutamide. On the other side of the spectrum, AutS decreased after treatment with bicalutamide or enzalutamide. These results support that the AutS is a reliable tool to distinguish patients with different susceptibilities to ADT. Although many immunohistochemical markers have been proposed for autophagy, the disadvantage they shared is instability and may be easily disturbed, which makes them unsuitable for clinical use [36]. AutS was generated based on six autophagy-related genes and revealed the intrinsic effects of different responses to ADT in PCa at the molecular level, indicating high stability and specificity. As far as we are concerned, AutS would be a promising method for selecting PCa patients sensitive to ADT, providing an essential tool for precise treatment and avoiding premature resistance to ADT.

We validated that PSA, GS, T stage, and AutS could be independent prognostic risk factors in PCa but excluded age. PSA, GS, T stage, and AutS were used as covariates, and the recurrence rate was used as a dependent variable. We designed a high-discrimination nomogram for the PCa recurrence probability prediction. Traditionally, PSA, GS, and T stage are fundamental for defining “high-risk” and “low-risk” PCa in different classification systems. In the National Comprehensive Cancer Network (NCCN), “high-risk” was defined as T3a, Gleason ≥8, or PSA ≥20 [37]; in the Radiation Therapy Oncology Group (RTOG), “high-risk” was defined as (1) Gleason ≥8 or (2) Gleason =7 plus either ≥ cT3 or node-positive [38]. Significant heterogeneity in outcomes predicted by different risk stratification schemes and 5-year relapse-free survival probabilities ranging from 49-80% were reported using different classifications for the same patient [39]. According to Chang et al., one of the reasons for this heterogeneity is that some groups used PSA and GS as continuous rather than dichotomous variables, resulting in a significantly different constitution of “high-risk” [40]. In our nomogram risk model, we considered PSA, GS, and T stage as dichotomous variables and gave them different weights based on their impacts on the prognosis of PCa. In addition, the dichotomous T stage can reduce the inherent inaccuracy for a 23% overstaging rate [41]. ROC analysis is a common method to assess the predictive accuracy of a risk model. However, it cannot reveal which models are more worthy of clinical use. For instance, a model with high specificity but low sensitivity would have a high AUC, whereas the false-negative rate is high at the same time, which would lead to a poorer outcome than those with a high false-positive rate [18]. Therefore, we tested the clinical value with extra DCA. Compared to PSA, GS, and T stage alone, the nomogram showed the highest discriminative accuracy and clinical net benefit. We believe it will be a promising tool for urologists to quantify the 3-year and 5-year recurrence rates and optimize treatment schemes.

To further investigate the underlying molecular mechanisms contributing to heterogeneity among the high-AutS and low-AutS groups, GSVA was conducted. We found different pathway activation between the two groups. In the high-AutS group, more proliferation-related pathways were enriched, including the G2 M checkpoint, E2F targets, mitotic spindle, MYC targets V1 and V2 pathways, mTORC1, and peroxisome and oxidative phosphorylation pathways. These signaling pathways have been proposed to promote cancer progression in diverse ways, involving regulation of the cell cycle, glucose metabolism, mitochondrial metabolism, protein synthesis, nucleotide synthesis, and lipid metabolism. In addition, we also examined the impact of the genetic alteration landscape on the prognosis of PCa. *TP53* is among the most frequent genomic alterations in late-stage PCa compared with early-stage PCa [42]. It was reported that 44% of patients with inherited *TP53* variants were diagnosed with high-PSA (1.1-171 ng/dl) and high-grade disease (Gleason ≥8) [43]. FOXA1 functions as a cofactor for AR and can promote tumor growth independently even in some AR deletion cases. *FOXA1* mutation is correlated with a more than 2-3-fold increase in growth, higher GS, and shorter relapse-free survival compared to the vector control [44]. KMT2D is an epigenetic modifier that enhances the expression of Kruppel-like factor-4 (KLF4) and leukemia inhibitory factor receptor (LIFR), which are involved in the activation of the PI3K/Akt and EMT pathways and promote the outgrowth and metastasis of PCa. Deletion of *KMT2D* results in DNA damage and cell apoptosis and senescence via the ROS pathway, thereby delaying the development of PCa [45, 46]. Obviously, high mutation frequencies of these genes in the high-AutS group explain the poor prognosis of high-AutS patients in our study.

Notably, *SPOP* mutation was confirmed as the driver event in PCa. *SPOP* mutation is reported in 5-15% of tumors, accounting for the most frequent point mutation in PCa [47]. However, opinions vary regarding its impact on the progression of PCa. Blattner M et al. considered no correlations between clinical outcome and *SPOP* mutation [48]. Others reported that *SPOP* mutation or decreased expression contributed to shorter OS and prevented the degradation of ERG and AR [49, 50]. In this study, we validated that SPOP mutation would downregulate its expression and lead to a shorter OS. Interestingly, SPOP is consistent with the level of AR activity, suggesting a good response to ADT, which was also observed in the high-AutS group.

Nevertheless, some shortcomings still exist in this study. We obtained autophagy genes from only three sources, and some appropriate genes may be missed. Our work was performed based on publicly available datasets, and more prospective cohorts from the real world were needed to test the model. We preliminarily revealed the different molecular mechanisms limited in transcriptomics, and further mechanistic studies may help better understand the different prognoses between the two groups.

## 5. Conclusion

Taken together, we constructed a prognostic autophagy-related gene signature for PCa. Its discriminative efficacy and clinical and therapeutic value were validated. The results of the mechanism analysis were consistent with the correlation study, proving the model reliability. In addition, a high-accuracy nomogram was also validated to quantitatively prognosticate the 3-year and 5-year recurrence rates of PCa. We believe it might be a promising risk stratification scheme for precisely personalized treatment for PCa patients.

## Figures and Tables

**Table 1:** Clinicopathological features among the four cohorts.

	TCGA_TCGA	GSE46602	GSE70768	MSKCC	Total
	(*n* = 431)	(*n* = 27)	(*n* = 108)	(*n* = 136)	(*n* = 702)
Age (years)					
Mean (SD)	60.9 (6.7)	62.5 (6.1)	60.4 (6.6)	58 (6.7)	60.4 (6.8)
Median (min, max)	61 (41, 78)	63 (46, 71)	62 (41, 73)	57.8 (37.3, 72.8)	61 (37.3, 78)
PSA (ng/ml)					
Mean (SD)	1 (4)	19.8 (10)	8.6 (3.7)	12.2 (44.3)	5.1 (20.6)
Stage					
Stage II	164 (38.1%)	13 (48.1%)	33 (30.6%)	85 (62.5%)	295 (42.0%)
Stage III	259 (60.1%)	14 (51.9%)	75 (69.4%)	44 (32.4%)	392 (55.8%)
Stage IV	8 (1.9%)			7 (5.1%)	15 (2.1%)
Gleason					
6	40 (9.3%)	9 (33.3%)	16 (14.8%)	40 (29.4%)	105 (15.0%)
7	213 (49.4%)	15 (55.6%)	84 (77.8%)	76 (55.9%)	388 (55.3%)
8	55 (12.8%)	2 (7.4%)	8 (7.4%)	10 (7.4%)	75 (10.7%)
9	120 (27.8%)	1 (3.7%)	0 (0.0%)	10 (7.4%)	131 (18.7%)
10	3 (0.7%)	0 (0.0%)	0 (0.0%)	0 (0.0%)	3 (0.4%)

**Table 2:** Chemo drugs for selected autophagy genes.

Drug	Symbol	Cor	Fdr	Drug	Symbol	Cor	Fdr
AR-42	CAPN10	-0.179	<0.001	MP470	CAPN10	-0.083	0.048
	NLRC4	-0.174	<0.001		NLRC4	-0.09	0.03
	UBE2T	-0.119	0.001		UBE2T	-0.115	0.006
Belinostat	CAPN10	-0.163	<0.001	Navitoclax	CAPN10	-0.232	<0.001
	NLRC4	-0.149	<0.001		NLRC4	-0.18	<0.001
	UBE2T	-0.092	0.012		UBE2T	-0.21	<0.001
BMS345541	CAPN10	-0.14	<0.001	NPK76-II-72-1	CAPN10	-0.213	<0.001
	NLRC4	-0.152	<0.001		NLRC4	-0.159	<0.001
	UBE2T	-0.091	0.011		UBE2T	-0.168	<0.001
BX-912	CAPN10	-0.163	<0.001	PHA-793887	CAPN10	-0.162	<0.001
	NLRC4	-0.205	<0.001		NLRC4	-0.215	<0.001
	UBE2T	-0.092	0.01		UBE2T	-0.082	0.02
CAY10603	CAPN10	-0.177	<0.001	PI-103	CAPN10	-0.135	<0.001
	NLRC4	-0.182	<0.001		NLRC4	-0.198	<0.001
	UBE2T	-0.096	0.007		UBE2T	-0.089	0.014
CP466722	CAPN10	-0.151	<0.001	PIK-93	CAPN10	-0.165	<0.001
	NLRC4	-0.144	<0.001		NLRC4	-0.197	<0.001
	UBE2T	-0.08	0.026		UBE2T	-0.075	0.037
CUDC-101	CAPN10	-0.147	<0.001	QL-X-138	CAPN10	-0.134	<0.001
	NLRC4	-0.17	<0.001		NLRC4	-0.168	<0.001
	UBE2T	-0.097	0.008		UBE2T	-0.089	0.014
CX-5461	CAPN10	-0.187	<0.001	QL-XI-92	CAPN10	-0.098	0.006
	NLRC4	-0.104	0.004		NLRC4	-0.205	<0.001
	UBE2T	-0.106	0.003		UBE2T	-0.075	0.04
FK866	CAPN10	-0.194	<0.001	THZ-2-102-1	CAPN10	-0.179	<0.001
	NLRC4	-0.185	<0.001		NLRC4	-0.174	<0.001
	UBE2T	-0.162	<0.001		UBE2T	-0.099	0.006
Genentech Cpd 10	CAPN10	-0.13	<0.001	TL-2-105	CAPN10	-0.111	0.002
	NLRC4	-0.109	0.002		NLRC4	-0.142	<0.001
	UBE2T	-0.1	0.006		UBE2T	-0.083	0.025
GSK1070916	CAPN10	-0.172	<0.001	TPCA-1	CAPN10	-0.173	<0.001
	NLRC4	-0.154	<0.001		NLRC4	-0.167	<0.001
	UBE2T	-0.152	<0.001		UBE2T	-0.074	0.038
GSK690693	CAPN10	-0.091	0.012	Vorinostat	CAPN10	-0.204	<0.001
	NLRC4	-0.103	0.004		NLRC4	-0.207	<0.001
	UBE2T	-0.1	0.006		UBE2T	-0.173	<0.001
I-BET-762	CAPN10	-0.211	<0.001	WZ3105	CAPN10	-0.177	<0.001
	NLRC4	-0.235	<0.001		NLRC4	-0.186	<0.001
	UBE2T	-0.087	0.014		UBE2T	-0.11	0.002
KIN001-102	CAPN10	-0.137	<0.001	YM201636	CAPN10	-0.15	<0.001
	NLRC4	-0.15	<0.001		NLRC4	-0.118	0.001
	UBE2T	-0.101	0.004		UBE2T	-0.078	0.033
Methotrexate	CAPN10	-0.213	<0.001				
	NLRC4	-0.21	<0.001				
	UBE2T	-0.078	0.036				

## Data Availability

All data used in this work can be acquired from the GDC portal (https://portal.gdc.cancer.gov/), Gene-Expression Omnibus (GEO; https://www.ncbi.nlm.nih.gov/geo/).

## Supplementary Materials

Supplementary Materials Supplementary Figure. Figure S1: A. Raw data for combined expression profile of GSE662, GSE70768, and MSKCC cohort. B. Combined expression data of GSE662, GSE70768, and MSKCC cohorts after batch effect removal. Figure S2: A. Correlation between AutS and OS in TCGA-PRAD cohort. B. Clinicopathological features distribution of PCa between high-AutS and low-AutS groups in TCGA-PRAD cohort. Figure S3: K-M analysis for autophagy-related gene signature in patients with same clinicopathological parameters in TCGA-PRAD cohort. Figure S4: K-M analysis for ULK1, CAPN10, FKBP5, UBE2T, NLRC4, and BNIP3L in GEO-combined cohort. Figure S5: K-M analysis for autophagy-related gene signature in patients with same clinicopathological parameters in GEO-combined cohort.
