# Supplementary material for: Signature for Prostate Cancer Based on Autophagy-Related Genes and a Nomogram for Quantitative Risk Stratification
Source: Dis Markers. 2022 Jul 7;2022:7598942. doi: 10.1155/2022/7598942 (PMC9293571; doi:10.1155/2022/7598942)

**Figure S1. A.** Raw data for combined expression profile of GSE662, GSE70768 and MSKCC cohort. **B.** Combined expression data of GSE662, GSE70768 and MSKCC cohorts after batch effect removal.


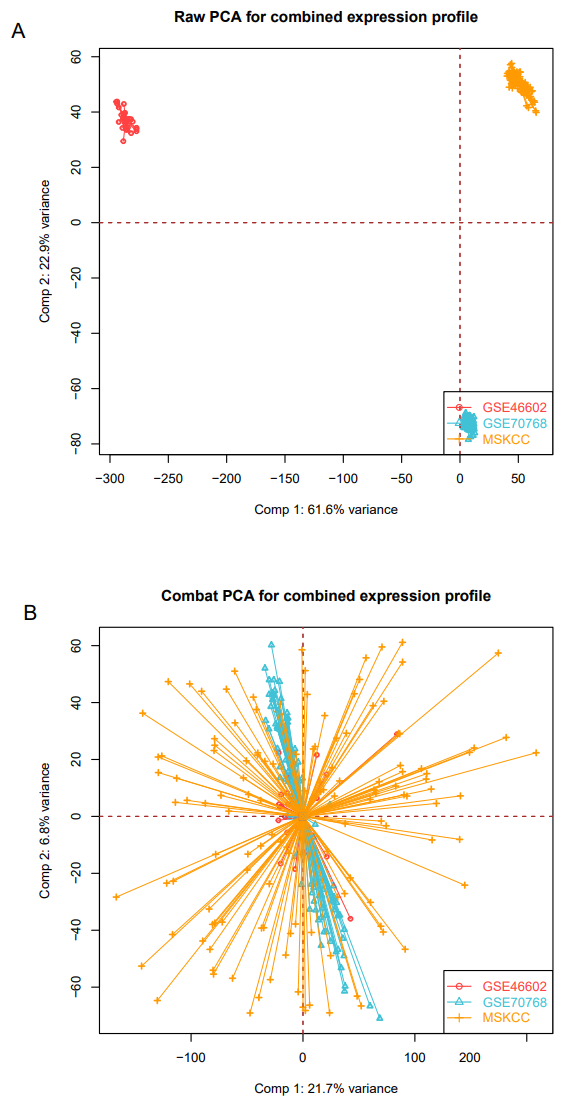


**Figure S2. A.** Correlation between AutS and OS in TCGA-PRAD cohort. **B.** Clinicopathological features distribution of PCa between high-AutS and low-AutS groups in TCGA-PRAD cohort.

**
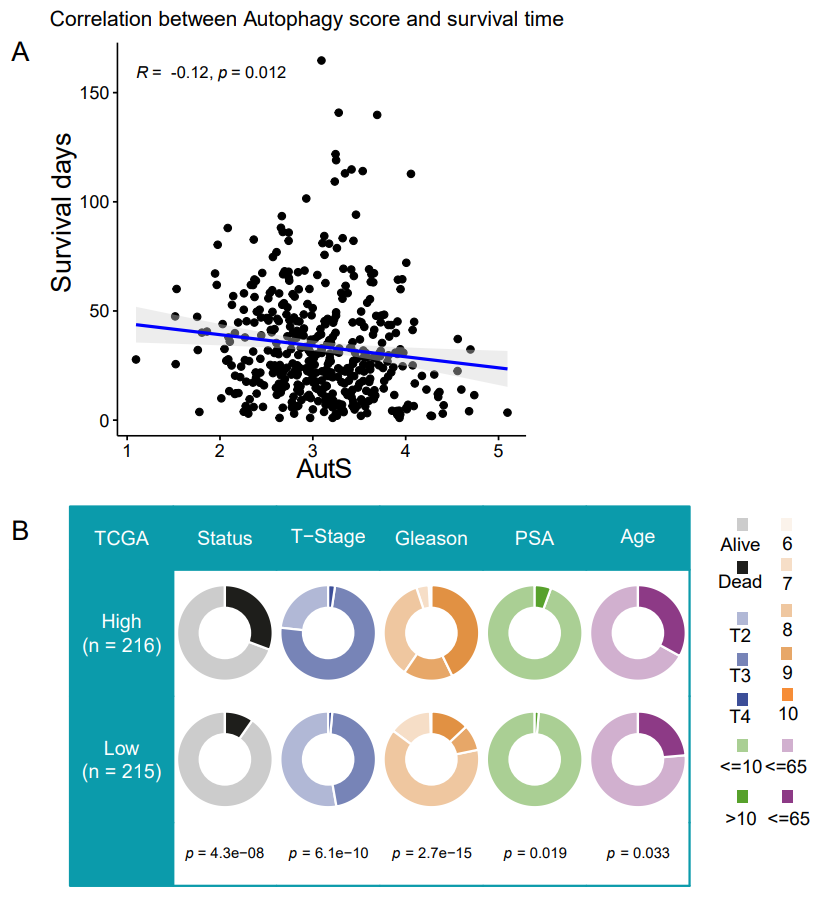
**

**Figure S3.** K-M analysis for autophagy-related gene signature in patients with same clinicopathological parameters in TCGA-PRAD cohort.


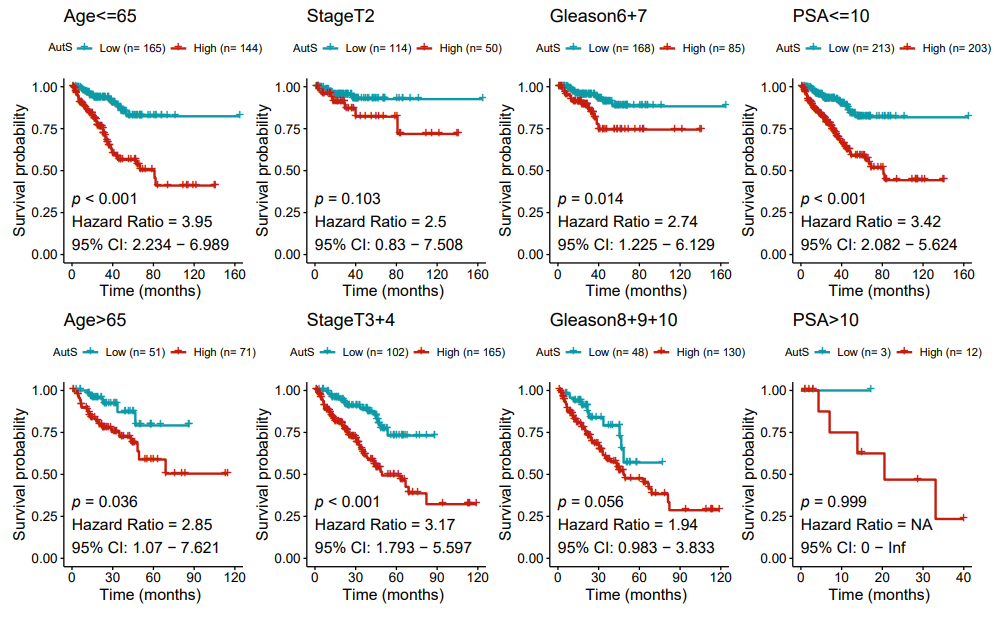


**Figure S4.** K-M analysis for ULK1, CAPN10, FKBP5, UBE2T, NLRC4 and BNIP3L in GEO-combined cohort.

**
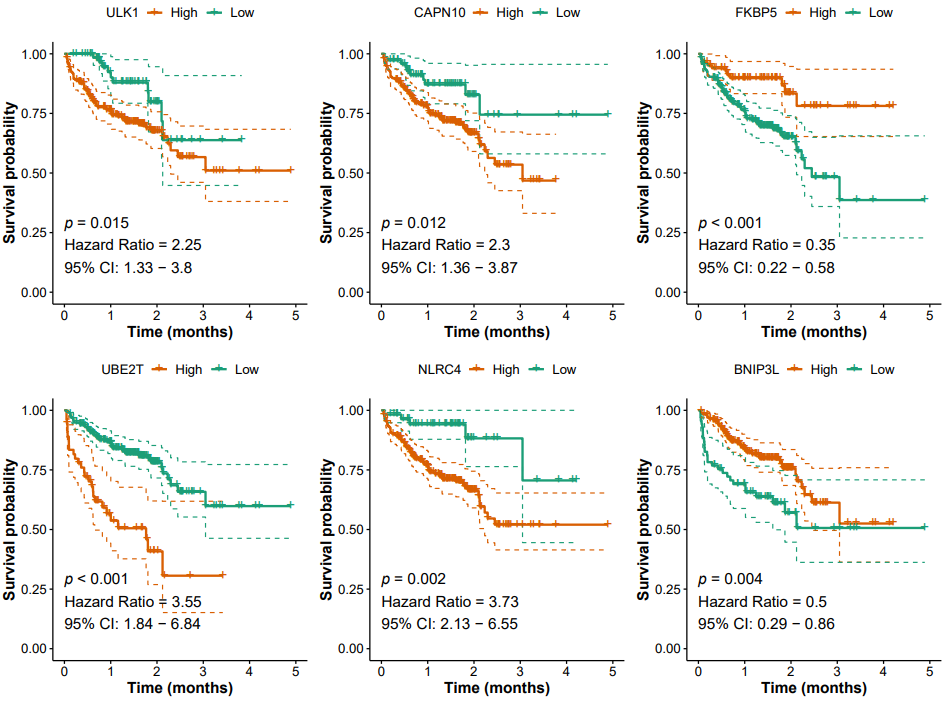
**

**Figure S5.** K-M analysis for autophagy-related gene signature in patients with same clinicopathological parameters in GEO-combined cohort.


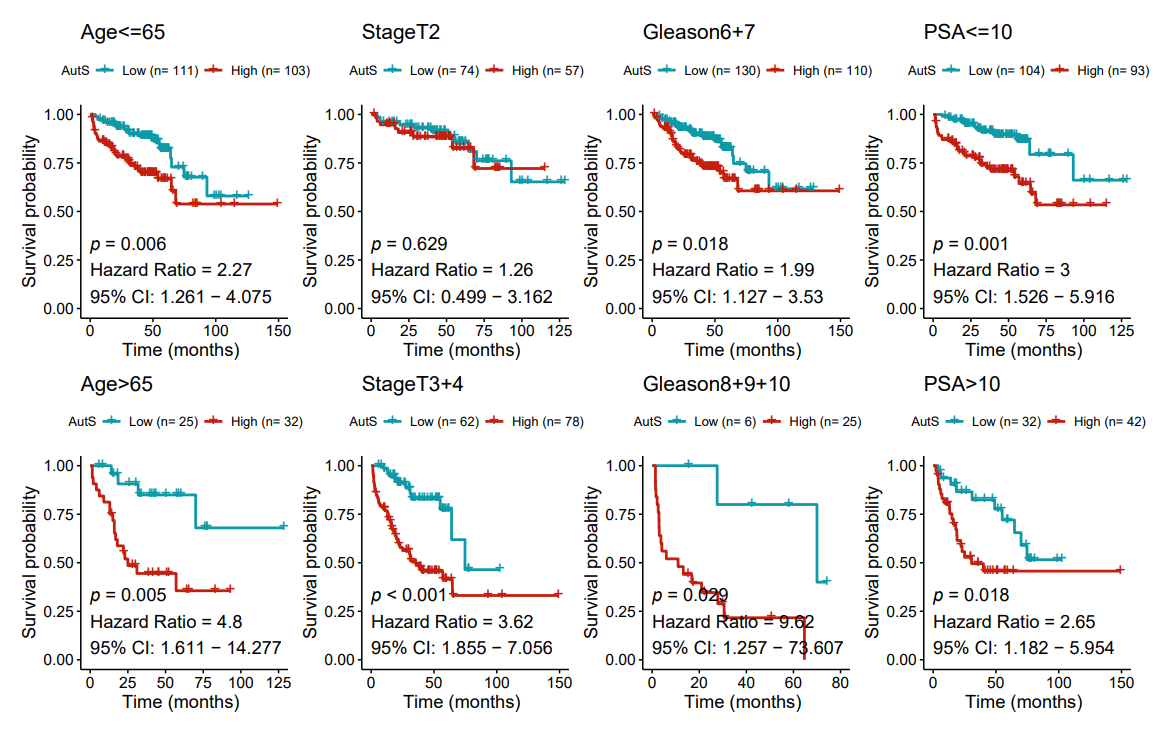

Supplement: Supplementary Materials — Supplementary Figure. Figure S1: A. Raw data for combined expression profile of GSE662, GSE70768, and MSKCC cohort. B. Combined expression data of GSE662, GSE70768, and MSKCC cohorts after batch effect removal. Figure S2: A. Correlation between AutS and OS in TCGA-PRAD cohort. B. Clinicopathological features distribution of PCa between high-AutS and low-AutS groups in TCGA-PRAD cohort. Figure S3: K-M analysis for autophagy-related gene signature in patients with same clinicopathological parameters in TCGA-PRAD cohort. Figure S4: K-M analysis for ULK1, CAPN10, FKBP5, UBE2T, NLRC4, and BNIP3L in GEO-combined cohort. Figure S5: K-M analysis for autophagy-related gene signature in patients with same clinicopathological parameters in GEO-combined cohort. [file 7598942.f1.docx]
